# Anti-HIV-1 Activity of the Integrase Strand Transfer Inhibitor ACC017

**DOI:** 10.3390/v18010033

**Published:** 2025-12-24

**Authors:** Meng-Di Ma, Rong-Hua Luo, Chun-Yan Li, Guan-Cheng Huang, Xin-Yan Long, Feng-Ying He, Liu-Meng Yang, He-Liang Fu, Yong-Tang Zheng

**Affiliations:** 1 State Key Laboratory of Genetic Evolution & Animal Models, Key Laboratory of Bioactive Peptides of Yunnan Province, KIZ-CUHK Joint Laboratory of Bioresources and Molecular Research in Common Diseases, Kunming Institute of Zoology, Chinese Academy of Sciences, Kunming 650223, China; mamengdi@mail.kiz.ac.cn (M.-D.M.); luorh@mail.kiz.ac.cn (R.-H.L.); lcyyanyan@126.com (C.-Y.L.); longxinyan@mail.kiz.ac.cn (X.-Y.L.); lmyang@mail.kiz.ac.cn (L.-M.Y.); 2 University of Chinese Academy of Sciences, Beijing 100049, China; 3 College of Pharmacy, Dali University, Dali 671000, China; 4 Chengdu Aidea Pharma-Tech Co., Ltd., Chengdu 610219, China; huanggc@aidea.com.cn (G.-C.H.); hefy@aidea.com.cn (F.-Y.H.); 5 Jiangsu Aidea Pharmaceutical Co., Ltd., Yangzhou 225008, China

**Keywords:** HIV-1, antiviral activity, INSTIs, ACC017, pharmacodynamics

## Abstract

HIV-1 integrase strand transfer inhibitors (INSTIs) are pivotal to antiretroviral therapy. However, the emergence of drug-resistant mutations necessitates the development of new agents. Here, we present ACC017 as a novel INSTI candidate. ACC017 demonstrated potent activity against the laboratory-adapted HIV-1_IIIB_ strain (EC_50_ = 0.59 nM; SI > 34,525) and maintained efficacy against a panel of drug-resistant strains (EC_50_ range from 0.34 to 9.12 nM) and clinical isolated strains (EC_50_ range from 0.11 to 1.78 nM). Mechanism of action studies confirmed its ability to inhibit the integrase enzyme (IC_50_ = 9.19 nM) and effectively block viral genome integration. Notably, in vitro resistance selection primarily yielded D232N and R263K mutations, without the emergence of G140S/A/C/R or Q148H/R/K. This promising profile, combined with synergistic interactions with other antiretroviral drugs, positions ACC017 as a potential therapeutic option.

## 1. Introduction

According to the data from the World Health Organization (WHO), 40.8 million people were living with human immunodeficiency virus (HIV) in 2024. Among them, 77% were able to receive antiretroviral therapy (ART) and 73% had their viral loads suppressed [1]. This indicates that antiretroviral treatment remains the primary and effective approach. Currently, the anti-HIV-1 drugs approved by the United States Food and Drug Administration (US FDA) are mainly classified into seven categories: nucleoside reverse transcriptase inhibitors (NRTIs), non-nucleoside reverse transcriptase inhibitors (NNRTIs), protease inhibitors (PIs), integrase strand transfer inhibitors (INSTIs), entry inhibitors, capsid inhibitor and pharmacokinetic enhancers [2,3]. Until 2007, when the first integrase inhibitor was approved by the US FDA [4], anti-HIV-1 drugs mainly targeted reverse transcriptase and protease. In 2021, WHO published new consolidated HIV guidelines and recommended the combination of dolutegravir (DTG) and a NRTI as the first-line antiretroviral drug regimen for people living with HIV initiating ART, and a raltegravir (RAL)-based regimen as the preferred first-line regimen for neonates. From this, INSTIs play an important role as key drugs in ART.

**Figure 1:**
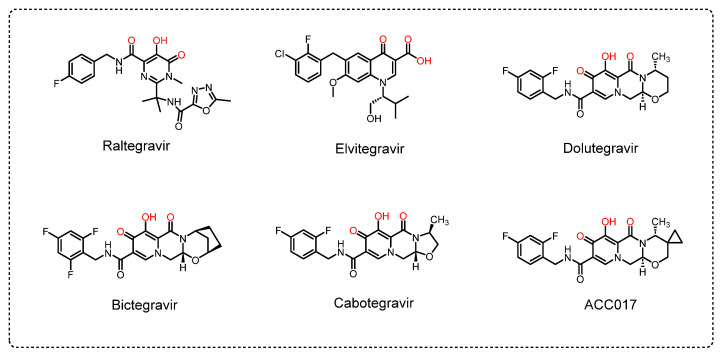
Chemical structures of HIV-1 INSTIs.

HIV-1 integrase is a 288 amino acid enzyme derived from the Gag-Pol viral polyprotein and is [5]. The integrase domain consists of the N-terminal domain I (NTD), catalytic core domain (CCD), and C-terminal domain (CTD) [6,7]. Integrase has become an important target in the design of anti-HIV-1 drugs. So far, there are five US FDA-approved INSTIs (Figure 1), including the first-generation drugs raltegravir (RAL) and elvitegravir (EVG) and the second-generation drugs dolutegravir (DTG), bictegravir (BIC) and cabotegravir (CAB). All of these are diketonic acid chain transfer inhibitors [8,9].

Although INSTIs have strong antiviral activity, a high selectivity index, and moderate toxicity, resistance to them is associated with mutations in HIV-1 integrase. The International Antiviral Society-USA list of resistance mutations has shown that the resistance sites G118R, E138A/K, G140A/C/S, Q148H/K/R and R263K are associated with resistance to all FDA-approved INSTIs [10]. In addition, mutations E138T, S153F/Y, G140R, N155H and T97K are detected as resistance mutations associated with some INSTIs [10,11,12,13,14,15,16]. Other mutation sites of RAL include L74M, E92Q, F121Y and Y143R/H/C [10,17,18,19]. Mutations S147G, F121Y, E92Q/G, and T66I/A/K are reported as resistance sites for EVG [10,20,21,22]. The first-generation INSTIs (RAL and EVG) have a lower resistance genetic barrier and a higher cross-resistance, so the current recommendation is to use the second-generation INSTIs (DTG, BIC and CAB) [10]. However, the reported side effects of DTG include nausea, occasional dizziness, birth defects, liver problems, and weight gain [23,24,25,26]. The most commonly reported side effects of BIC are diarrhea, headache, nausea, and tiredness [23,27]. The most frequently reported adverse events of CAB include fever, headache, nausea and fatigue [23,28]. Therefore, there is still a need to develop anti-HIV-1 drugs with better antiviral effects, lower toxic side effects, and higher drug-resistance barriers. In this study, we described a new compound, ACC017, as an integrase chain transfer inhibitor with antiviral activity comparable to that of the control drug DTG, which is expected to be developed as a novel anti-HIV-1 drug.

## 2. Materials and Methods

### 2.1. Ethics Statement

The ethical approval and consent process of this study was provided by the Ethics Committee of Kunming Institute of Zoology, Chinese Academy of Sciences (Approval Number: KIZRKX-2021-013).

### 2.2. Compounds

ACC017, Lamivudine (3TC), Emtricitabine (FTC), Tenofovir alafenamide (TAF), Ainuovirine (ACC007), and Dolutegravir (DTG) were provided by Jiangsu Aidea Pharmaceutical Co., Ltd. (Yangzhou, China). Efavirenz (EFV) was purchased from MedChemExpress (MCE, Monmouth Junction, NJ, USA). FTC was dissolved in 0.9% sodium chloride injection (Sigma-Aldrich, St. Louis, MO, USA) and stored at −20 °C. Other compounds were dissolved in dimethyl sulfoxide (DMSO, MCE) and stored at 4 °C.

### 2.3. Cells and Viruses

C8166 and MT-4 cells were provided by the AIDS Reagent Project of the UK Medical Research Council (MRC, London, UK). Human peripheral blood mononuclear cells (PBMCs) were purchased from Shanghai Heyousheng Biotechnology Co., Ltd. (Shanghai, China). All cells were cultured in RPMI-1640 medium (Gibco, Waltham, MA, USA) containing 10% fetal bovine serum (Gibco), 100 units/mL penicillin (Sigma-Aldrich) and streptomycin (Amresco, Solon, OH, USA). For PBMCs activation, cells pooled from multiple donors were stimulated with 5 µg/mL phytohemagglutinin (PHA; Sigma-Aldrich) and 50 IU/mL recombinant human interleukin-2 (IL-2; Meilunbio^®^, Dalian, China). Stock solutions of PHA and IL-2 were prepared in advance, aliquoted, and stored at −80 °C. Following 72 h of activation, cells were harvested, and viable cell density was uniformly adjusted using absolute counting on a BD LSR Fortessa flow cytometer (Franklin Lakes, NJ, USA) prior to HIV-1 infection.

Laboratory-adapted strain HIV-1_IIIB_ was provided by the AIDS Reagent Project of the UK Medical Research Council (MRC). NRTI-resistant mutation strain HIV-1_4755-5_, NNRTI-resistant mutation strain HIV-1_A17_, FI-resistant mutation strain pNL4-3_GP41(36G) V38A, N42T_, and PI-resistant mutation strain HIV-1_RF/V82F/184V_ were obtained from the NIH AIDS Research and Reference Reagent Program (Rockville, MD, USA) The INSTI-resistant mutant strain pNL4-3_G140S/Q148H_ was constructed in the laboratory using a site-directed mutagenesis kit (TransGen Biotech, Beijing, China). Clinical isolated HIV-1 strains from AIDS patients in Yunnan, China were co-cultured with human healthy PBMCs, including HIV-1_TC-1_ (CRF01_AE), HIV-1_KIZ011_ (CRF07_BC), and HIV-1_WAN_ (CRF01_AE). All virus stocks were equally distributed and stored at −80 °C.

### 2.4. Cytotoxicity Assays

The cytotoxicity of ACC017 was determined by the MTT assay as described previously [29], with DTG as a positive control. C8166, MT4 cells (4 × 10^5^/mL) or PBMCs (5 × 10^6^/mL) were seeded in 96-well plates, and gradient-diluted compounds were added. After incubating for 3 d (7 d for PBMCs) at 37 °C, 5% CO_2_, 20 μL of MTT (3-(4,5-dimethyl-2-thiazolyl)-2,5-diphenyl-2-H-tetrazolium bromide, Thiazolyl Blue Tetrazolium Bromide) (Sigma-Aldrich) was added to each well for 4 h. Then, 100 μL of cell supernatants were discarded, and the cells were treated with 100 μL of 12% sodium dodecyl sulfate-50% N, N-dimethylformamide (DMF) (Sigma-Aldrich) at 37 °C overnight. The absorbance was measured using a Bio Tek 800TS (Agilent Technologies, Santa Clara, CA, USA) at 570/630 nm, and the 50% cytotoxicity concentration (CC_50_) was calculated.

### 2.5. Anti-HIV-1 Activities Assay

The efficacy of ACC017 against HIV-1_IIIB_ was evaluated in C8166 cells based on the viral-induced cytopathic effect (CPE), with DTG used as a positive control. HIV-1_IIIB_ (multiplicity of infection = 0.01) and C8166 cells (4 × 10^5^/mL) were co-cultured with gradient-diluted compounds for 3 d. Syncytia were counted under an inverted microscope (100×), and the 50% effective concentration (EC_50_) was calculated using GraphPad Prism 8.0 (GraphPad Software, San Diego, CA, USA).

The inhibitory activity of ACC017 against a laboratory-adapted strain, drug-resistant strains and clinical isolated strains was detected using the HIV-1 p24 antigen capture assay as mentioned earlier [30,31]. In 96-well plates with gradient-diluted drugs, C8166 cells (4 × 10^5^/mL) were infected with HIV-1_IIIB_, HIV-1_4755-5_, HIV-1_A17_, pNL4-3_GP41(36G) V38A, N42T_, HIV-1_RF/V82F/184V_ or pNL4-3_G140S/Q148H_ (MOI = 0.05), and PBMCs (2.5 × 10^6^/mL) were infected with HIV-1_TC-1_, HIV-1_WAN_ or HIV-1_KIZ011_ (MOI = 0.1). After incubation at 37 °C for 4 h, the cells were washed three times with PBS to remove free viruses. The resuspended cells were cultured with gradient-diluted compounds for 4 d (C8166) or 7 d (PBMCs). For human serum effect evaluation, C8166 cells infected with HIV-1_IIIB_ were co-cultured with diluted compounds in medium with or without 20% human serum for 4 d. The anti-HIV-1 activity of ACC017 was evaluated by measuring HIV-1 p24 antigen levels in culture supernatants using ELISA. Absorbance measurements at 490/630 nm were made using a Bio Tek 800TS, and the EC_50_ value was calculated. The anti-HIV-1_IIIB_ activity of ACC017 in MT-4 (4 × 10^5^/mL) cells was determined based on the aforementioned MTT assay, and the EC_50_ value was calculated. Each assay included DTG as a positive control. The EC_50_ was calculated using GraphPad Prism 8.0.

### 2.6. Combined Antiviral Activity Assay

The anti-HIV-1 effects of ACC017 in combination with NRTIs (3TC, or FTC and TAF), an NNRTI (ACC007), and INSTIs (DTG or ACC017) were detected in C8166 cells infected with HIV-1_IIIB_. Different drug gradients were used based on the EC_50_ value of each drug alone for inhibiting HIV-1 replication. The level of HIV-1 p24 in the culture supernatants was determined by ELISA. The dose reduction index (DRI) and combination index (CI) were calculated according to the median effect principle using CompuSyn software version 1.0 (ComboSyn Inc., Paramus, NJ, USA) [32,33]. CI > 1.1 = antagonism, 0.9–1.1 = additive, <0.9 = synergism. The CI value of the drug combination was calculated by the following formula:

CI value = (1 × ED_50_ + 2 × ED_75_ + 3 × ED_90_ + 4 × ED_95_)/10

### 2.7. In Vitro HIV-1 Integrase Inhibitory Assay

The assay was performed in accordance with the instructions of the EZ-1700/EZ-1800 HIV-1 Integrase Assay Kit (Xpressbio, Frederick, MD, USA). Briefly, DS DNA was incubated in a microplate for 1 h and blocking buffer was incubated for 0.5 h at 37 °C. Then, HIV-1 full-length recombinant integrase was added and incubated for 0.5 h at 37 °C. Subsequently, the gradient-diluted drugs were added to the microplate and incubated at room temperature for 5 min. The TS DNA and HRP reaction solutions were added sequentially to the microplate, and each was incubated for 0.5 h. A TMB peroxidase substrate solution was added to each well and incubated for 10 min at room temperature. The absorbance values were read at 450 nm. The half-maximal inhibitory concentration (IC_50_) was calculated using GraphPad Prism 8.0.

### 2.8. Molecular Docking Assay

Docking simulations were performed using the Schrödinger Suite (version 2021-2; Schrödinger, LLC, New York, NY, USA). The crystal structure of HIV-1 integrase (PDB: 6PUW) was prepared using the Protein Preparation Wizard, and ligands were processed with LigPrep. Glide was used for docking in Standard Precision (SP) mode, with the receptor grid centered on the active site (143.7, 159.32, 178.94). Default parameters were applied for flexible ligand docking, and interaction analysis was conducted using Maestro to identify aromatic hydrogen bonds, salt bridges, and π–π stacking interactions.

### 2.9. Determination of Late-RT, 2LTR and Alu-LTR

1 × 10^6^/mL C8166 cells were co-incubated with HIV-1_IIIB_ (MOI = 0.5) at 4 °C for 2 h, washed twice with PBS to remove free viruses, then seeded in 24-well plates, and 100 nM ACC017 was added, with DTG used as a positive control. The infected cells were harvested at 2 h, 4 h, 6 h, 8 h, 12 h and 24 h, and DNA was extracted. Total genomic DNA was extracted using a Blood Genomic DNA Mini Kit (CWBIO, Beijing, China) and quantified. Serial dilutions of the Late-RT, 2-LTR, and Alu-LTR standards were prepared, and quantitative PCR was performed using the Premix Ex Taq™ (Probe qPCR) reagent (Takara Bio Inc., Kusatsu, Japan). A standard curve was generated to quantify Late-RT, 2-LTR, and Alu-LTR products.

Quantitative detection of late-RT (5′-TGTGTGCCCGTCTGTTGTGT-3′(F), 5′-GAGTCCTGCGTCGAGAGATC-3′(R), 5′-FAM-CAGTGGCGCCCGAACAGGGA-TAMRA-3′(probe)) and 2-LTR (5′-GCCTGGGAGCTCTCTGGCTAA-3′(F), 5′-AGGTAGCCTTGTGTGTGGTAGATCC-3′(R), 5′-FAM-TAGTGTGTGCCCGTCTGTTGTGTGAC-TAMRA-3′(probe)) using the QuantStudio 5 Real-Time PCR System (Applied Biosystems, Waltham, MA, USA). PCR reactions were carried out with a holding stage of 2 min at 95 °C followed by 40 cycles of 15 s at 95 °C, 60 °C for 30 s. The expression levels of late-RT and 2-LTR were analyzed according to standard curves.

The nested PCR method was used to detect Alu-LTR at 24 h after infection. The sequences of the primers used in the first round of PCR amplification were as follows:

L-M667 5′-ATGCCACGTAAGCGAAACTCTGGCTAACTAGGGAACCCACTG-3′.

Alu1 5′-TCCCAGCTACTGGGGAGGCTGAGG-3′.

Alu2 5′-GCCTCCCAAAGTGCTGGGATTACAG-3′.

PCR reactions were carried out with a holding stage of 8 min at 95 °C followed by 12 cycles of 10 s at 95 °C, 60 °C for 10 s, 72 °C for 170 s.

The second round of PCR used the first amplification product as a template and primers were used:

AA55M 5′-GCTAGAGATTTTCCACACTGACTAA-3′.

LambdaT 5′-ATGCCACGTAAGCGAAACT-3′.

probe-LTR 5′-TAGTGTGTGCCCGTCTGTTGTGTGAC-3′.

PCR reactions were carried out with 8 min at 95 °C followed by 50 cycles of 10 s at 95 °C, 60 °C for 30 s.

### 2.10. In Vitro Selection of HIV-1 Mutant Strains and Analysis of Drug Resistance

HIV-1 strains resistant to ACC017 were obtained after sequential passaging of HIV-1_IIIB_ in the presence of increasing concentrations of ACC017 in C8166 cells. At the start of the selection, 1 × 10^6^/mL C8166 cells were inoculated with the HIV-1_IIIB_ (MOI = 0.05) in the presence of a 1 nM concentration of ACC017. From the fourth day on, cell morphology was observed daily. When the cytopathic effect (CPE) appeared in 80% of the cells, the culture supernatant was centrifuged, aliquoted, and stored frozen at −70 °C. This was regarded as one passage. Then, 1 mL of the virus supernatant was taken to infect new C8166 cells, and twice the previous concentration of ACC017 was added. After more than six months of drug induction and serial passaging (13 passages), we were able to culture resistant virus (HIV-1_DRACC017_) in the presence of 4096 nM of ACC017. In a parallel control experiment, HIV-1_IIIB_ was cultured in C8166 cells in the absence of ACC017.

Viral RNA was extracted using the High Pure Viral RNA Kit (Roche Diagnostics, Basel, Switzerland) according to the manufacturer’s protocol. Reverse transcription was performed with the PrimeScript™ II 1st Strand cDNA Synthesis Kit (Takara), followed by polymerase chain reaction using TransTaq^®^ DNA Polymerase High Fidelity (Takara) and the primers set 5′-GCATGGGTACCAGCACACAAAG-3′ (F) and 5′-CTAGCTTTCCCTGAAACATACATATGGTG-3′ (R). The resulting amplicons were purified and ligated into a cloning vector, which was then transformed into competent cells. Positive clones, confirmed by electrophoresis, were inoculated into LB liquid medium and subjected to overnight shaking culture. These cultures were sent for sequencing, with a minimum of 30 clones successfully sequenced. The obtained sequences were aligned and compared against the HIV-1 reference sequence (GenBank: NC_001802) using MEGA 11.0 software. For quality control during the induction of drug-resistant strains, the concurrently cultured negative control HIV-1_IIIB_ was also sequenced and analyzed.

The mutant plasmid NL4-3_IN-R263K_ was constructed using the Fast Mutagenesis System kit (TransGen Biotech) with the primers 5′-GCATGGGTACCAGCACACAAAG-3′ (F) and 5′-CTAGCTTTCCCTGAAACATACATATGGTG-3′ (R). The viruses were packaged and amplified separately in 293T and C8166 cells. The inhibitory activity of ACC017 against HIV-1_DRACC017_ (MOI = 0.1) and NL4-3_IN-R263K_ (MOI = 0.1) was detected using the HIV-1 p24 antigen capture assay. Each assay included DTG, 3TC and EFV as drug controls. The EC_50_ was calculated using GraphPad Prism 8.0.

### 2.11. Data Statistics

The data in this study represented three independent repetitions of each experiment and are shown as the mean ± standard deviation (SD). Statistical significance was assessed using One-way ANOVA analysis was performed to determine the *p* value. * *p* < 0.05, ** *p* < 0.01, and *** *p* < 0.001 denotes a significant difference.

## 3. Results and Discussion

### 3.1. Activity of ACC017 Against HIV-1

**Figure 2:**
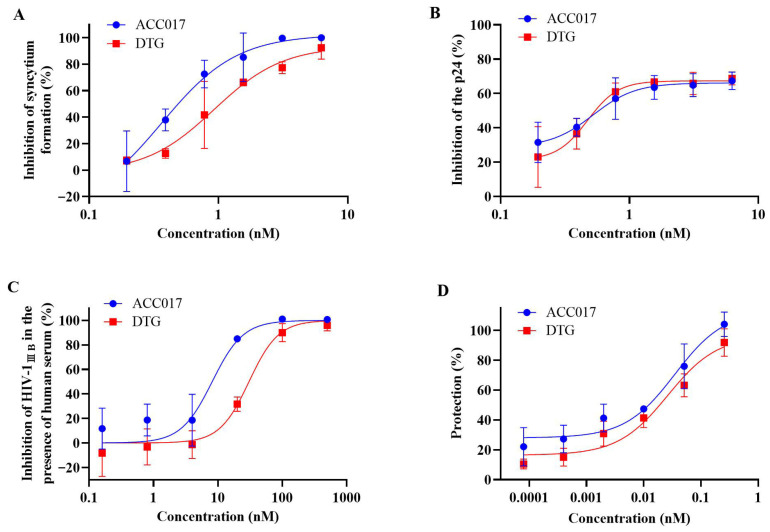
Activity against HIV-1_IIIB_. The cytopathic effect was tested by counting the number of syncytia (**A**). The antiviral effect of ACC017 against HIV-1_IIIB_ was determined by detecting HIV-1 p24 antigen using ELISA (**B**), and the antiviral effect of ACC017 against HIV-1_IIIB_ in the presence of 20% human serum was also determined (**C**). The protective effect of ACC017 was measured in the MT-4 cell line by MTT assay (**D**). DTG used as a positive control. All data represent as mean ± SD (n = 3).

ACC017 was tested for anti-HIV-1 activity in C8166, MT-4 and PBMC cells were infected with different HIV-1 strains and was compared with DTG. The results are presented as EC_50_, CC_50_ and SI (Table 1). ACC017 inhibited HIV-1_IIIB_-induced cytopathy in C8166 cells, with potent activity (EC_50_ = 0.59 ± 0.09 nM), low cytotoxicity (CC_50_ = 20.37 ± 0.10 µM) and a high selectivity index (SI > 34,525.42), comparable to DTG (EC_50_ = 0.73 ± 0.02 nM, CC_50_ = 20.34 ± 0.07 µM, SI > 27,863.01) (Figure 2A). In C8166 cells acutely infected with HIV-1_IIIB_, ACC017 had an EC_50_ of 0.59 ± 0.09 nM, also comparable to DTG (EC_50_ = 0.64 ± 0.07 nM) (Figure 2B).

Studies have shown that plasma proteins significantly affect the antiviral activity of drugs in vitro [34]. The FDA also recommended that researchers evaluate the antiviral activity of drugs in the presence of human serum. Therefore, the anti-HIV-1_IIIB_ activity of ACC017 was evaluated in the presence of 20% human serum. The results showed that serum proteins weakened the antiviral potency of both ACC017 and DTG. The EC_50_ of ACC017 increased 14.03-fold from 0.59 nM to 8.28 nM, while that of DTG increased 52.45-fold from 0.64 nM to 33.57 nM (Figure 2A,C). These findings indicate that the anti-HIV-1 activity of both compounds is susceptible to serum protein binding, with the effect being more pronounced for DTG in the presence of 20% human serum.

Another type of T lymphoid cell was used to test the antiviral effects of ACC017. MT-4 cells infected with HIV-1 die within 6–7 d. We found that ACC017 had a good protective effect against HIV-1_IIIB_ infection-induced MT-4 cell death. The EC_50_ was 51.47 ± 0.02 pM, which was comparable to that of DTG (EC_50_ = 51.44 ± 0.14 pM) (Table 1, Figure 2D).

**Figure 3:**
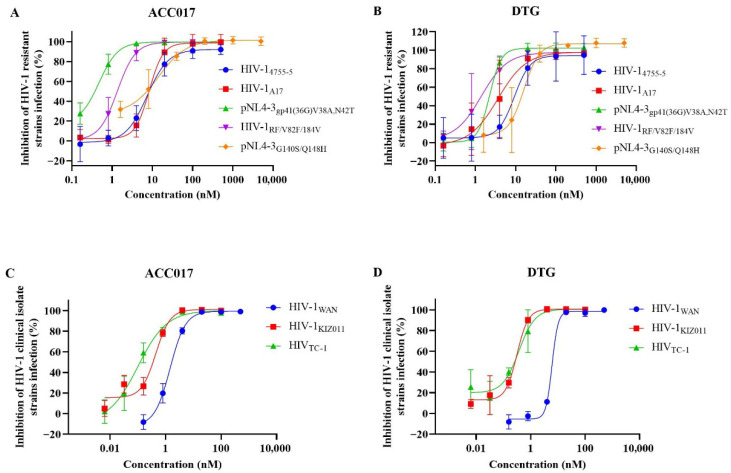
Activity against drug-resistant strains and clinical isolated strains. The antiviral effect of ACC017 and DTG on drug-resistant strains (**A**,**B**), and on HIV-1 clinical isolated strains (**C**,**D**) were determined by detecting HIV-1 p24 antigen using ELISA. All data represent as mean ± SD (n = 3).

We also evaluated the antiviral activity of ACC017 against drug-resistant strains (HIV-1_A17_, HIV-1_4755-5_, pNL4-3_gp41(36G) V38A, N42T_, HIV-1_RF/V82F/184V_ and pNL4-3_G140S/Q148H_) in C8166 cells. As shown in Table 1, ACC017 exhibited high potency against these strains (Figure 3A), with DTG serving as the control drug (Figure 3B). The EC_50_ values of ACC017 against the NRTI-resistant strain HIV-1_4755-5_ and the PI-resistant strain HIV-1_RF/V82F/184V_ were 9.12 and 1.45 nM, respectively, showing antiviral activity equivalent to that of DTG (EC_50_ = 9.96 and 1.69 nM, respectively). ACC017 showed inferior activity against the NNRTI-resistant strain HIV-1_A17_ (EC_50_ = 8.51 nM) compared with DTG (EC_50_ = 4.78 nM), but superior activity against the FI-resistant strain pNL4-3_gp41(36G) V38A, N42T_ (EC_50_ = 0.34 nM vs. 1.95 nM for DTG). Our experiments utilized a multi-cycle replication assay. In this context, the compensatory adaptations developed by the NRTI/NNRTI-resistant strain to maintain fitness may have unexpectedly increased its susceptibility to INSTIs. The enhanced potency of ACC017 against this resistant background highlights its lack of cross-resistance. In particular, ACC017 and DTG also exhibited strong inhibitory effects on the INSTI-resistant strain pNL4-3_G140S/Q148H_, with EC_50_ values of 8.19 nM and 13.46 nM, respectively. However, compared to their activity against the laboratory-adapted strain HIV-1_IIIB_, the susceptibility of the pNL4-3_G140S/Q148H_ strain to ACC017 decreased by 13.88-fold (EC_50_ from 0.59 nM to 8.19 nM), and to DTG by 21.03-fold (EC_50_ from 0.64 nM to 13.46 nM). This indicates that despite the reduced susceptibility of the HIV-1 integrase double mutant G140S/Q148H to ACC017 and DTG, both drugs remain effective in inhibiting viral replication.

After confirming the anti-HIV-1 activity of ACC017 against laboratory-adapted strain and drug-resistant strains, further testing was conducted on the antiviral effect of ACC017 on HIV-1 clinical isolated strains, including HIV-1_KIZ011_ (CRF07_BC), HIV-1_TC-1_ (CRF01_AE), and HIV-1_WAN_ (CRF01_AE). ACC017 had a good inhibitory effect on HIV-1_WAN_, HIV-1_KIZ011_, and HIV-1_TC-1_ in PBMC cells, with EC_50_ values of 1.78 nM, 0.33 nM, and 0.11 nM, respectively (Table 1, Figure 3C). The antiviral activity of DTG against HIV-1_KIZ011_ (EC_50_ = 0.28 nM) and HIV-1_TC-1_ (EC_50_ = 0.31 nM) was comparable to that of ACC017 (Table 1, Figure 3D). However, for HIV-1 _WAN_, the EC_50_ value of DTG (EC_50_ = 8.23 nM) was about 4.62 times higher than that of ACC017 (EC_50_ = 1.78 nM). Meanwhile, ACC017 and DTG showed lower cytotoxicity in human PBMCs, with CC_50_ values of 15.24 µM and 14.56 µM, respectively. These results suggested that ACC017 had potential antiviral activity against clinical isolated strains of HIV-1.

### 3.2. Inhibitory Activity Against HIV-1 Integrase

The inhibitory effect of ACC017 on wild-type (WT) HIV-1 integrase and HIV-1 mutant N155H integrase was investigated by enzyme activity assay, with DTG used as a control drug. As shown in Table 2, ACC017 exhibited good inhibitory activities against WT HIV-1 integrase (IC_50_ = 9.19 nM) and HIV-1 mutant N155H integrase (IC_50_ = 9.91 nM), comparable to those of DTG (IC_50_ = 13.60 nM and 9.26 nM, respectively). This result indicated that ACC017 targeted HIV-1 integrase, which functions as a classical INSTI.

### 3.3. Molecular Docking Study

**Figure 4:**
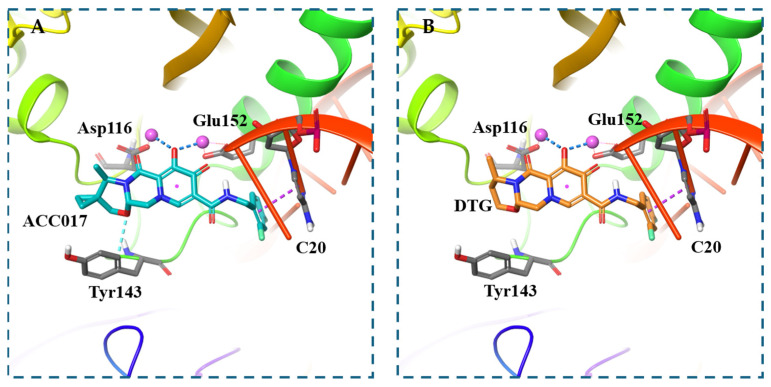
Molecular docking. Predicted binding mode of ACC017 with wild-type HIV-1 integrase (PDB ID: 6PUW) (**A**), Predicted binding mode of DTG with wild-type HIV-1 integrase (PDB ID: 6PUW) (**B**). Aromatic hydrogen bonds are shown as light blue dashed lines, salt bridges as deep blue dashed lines, and π–π stacking interactions as purple dashed lines.

To further explain the antiviral activity results of ACC017 and to better understand its binding mode, molecular docking was conducted. Docking simulations revealed distinct binding profiles for ACC017 and DTG within the active site of wild-type HIV-1 integrase. ACC017 formed a network of interactions including aromatic hydrogen bonding with Tyr143, two salt bridges with Glu152 and Asp116, and π–π stacking with the DNA base C20 (Figure 4A). In contrast, DTG exhibited fewer stabilizing interactions, primarily engaging in two salt bridges with Glu152 and Asp116 and π–π stacking (Figure 4B). The spatial orientation of both compounds around the C20 position highlighted differential engagement with key catalytic residues, suggesting that ACC017 may offer enhanced binding stability compared to DTG.

### 3.4. Antiviral Activity of ACC017 in Combination with Anti-HIV-1 Drugs

To assess the efficacy of ACC017 in combination with marketed anti-HIV-1 drugs, combination studies of ACC017 and other anti-HIV-1 drugs were conducted. This experiment adopted the principle of Zhou’s medium effect model to design a combined experimental medication plan and process data [31]. As shown in Table 3, the dosage of marketed anti-HIV-1 drugs (NRTIs and NNRTI) when combined with ACC017 was lower than that of the drugs alone (DRI = 2.41–17.41). The higher the DRI value, the greater the reduction in the drug dosage during combination therapy. This reduces the occurrence of corresponding drug side effects and slows down the emergence of resistant viruses. When ACC017 was combined with NRTIs/NNRTI, all had CI values below 0.9, indicating a synergistic effect between the drugs. Smaller CI values indicated stronger synergistic effects among the drugs. These results suggested that the combination of ACC017 with NRTIs or NNRTIs could be considered an alternative drug combination for antiretroviral therapy.

### 3.5. Effects of ACC017 on Formation of Late-RT, 2-LTR and Alu-LTR

The inhibitory efficacy of ACC017 on HIV-1 replication intermediates was evaluated by quantifying late reverse transcripts (Late-RT), 2-LTR circles (a surrogate for nuclear import), and integrated proviruses (Alu-LTR) using real-time quantitative PCR. To enhance the sensitivity and specificity of the integrated DNA detection, a nested Alu-LTR PCR assay was employed. The first-round PCR amplified the DNA fragment between the HIV-1 provirus DNA and the closest Alu element. The second-round real-time PCR quantified the integrated viral DNA copies.

**Figure 5:**
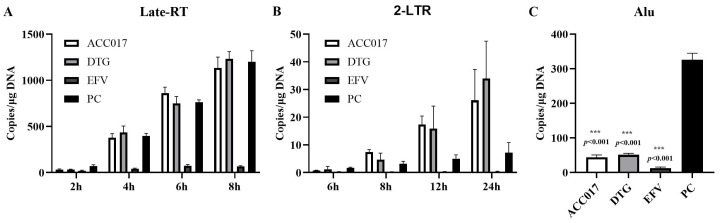
Effects of ACC017 on the formation of Late-RT (**A**), 2-LTR (**B**) and Alu-LTR (**C**) were investigated. The NNRTI-EFV (Efavirenz, an HIV-1 reverse transcriptase inhibitor), and the INSTI-DTG (Dolutegravir, an integrase strand transfer inhibitor) were used as positive drug controls. For the positive control (PC), C8166 cells were only infected with HIV-1_IIIB_ without drug treatment. Data are presented as mean ± SD (n = 3). One-way ANOVA analysis was performed to determine the *p* value. *** *p* < 0.001 denotes a significant difference.

As shown in Figure 5A, after viral infection, the Late-RT levels in the positive control (PC) group increased significantly at 4 h, 6 h and 8 h. This indicates that the double-stranded viral DNA produced by reverse transcription continued to increase. In the ACC017 and DTG treatment groups, Late-RT increased over time and showed no significant difference compared with the PC group, implying that ACC017 and DTG did not have an inhibitory effect on the HIV-1 reverse transcription process. The Late-RT level in the EFV treatment group was significantly lower than that in the PC group, suggesting that EFV suppressed the viral reverse transcription process. At 8 h, 12 h and 24 h after viral infection, 2-LTR in the PC group could be detected, reflecting that the viral DNA had entered the nucleus. In the ACC017 and DTG treatment groups, the level of 2-LTR increased over time, revealing that ACC017 and DTG were unable to block the nuclear entry process (Figure 5B). The Late-RT results showed that the EFV treatment group blocked the process of HIV-1 reverse transcription, and thus 2-LTR was barely detected within 24 h. Alu-LTR was detected in the PC group 24 h after viral infection, proving that the viral DNA had been integrated into the host genome by this time. Alu-LTR was significantly reduced in both the ACC017 and DTG treatment groups, suggesting that ACC017 and DTG blocked the HIV-1 genome integration process (Figure 5C). Alu-LTR was almost undetectable in the EFV treatment group because reverse transcription was blocked at an early stage of viral replication. ACC017 acted during the phase when the viral genome was being integrated into the host DNA, effectively blocking its integration. The mechanism of action of ACC017 was similar to that of the positive control drug DTG.

### 3.6. In Vitro Induction of ACC017-Resistant Strains and Analysis of Drug Resistance

Since drug resistance selection is one of the key aspects to be addressed in the process of developing new anti-HIV-1 drugs, resistant strains induced by ACC017 were detected, and phenotypic and genotypic identifications were conducted.

**Figure 6:**
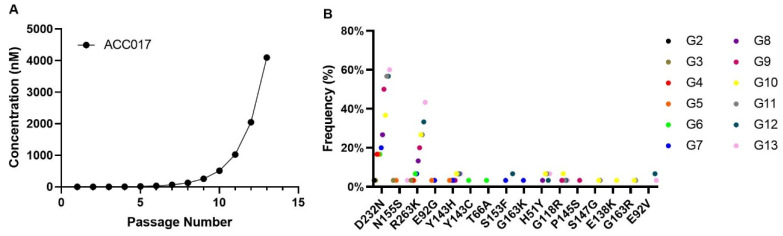
Selection of HIV-1_IIIB_-derived mutant strains under selective pressure and study of the drug resistance of ACC017. Kinetics of resistance development for HIV-1_IIIB_ under selective pressure with compound ACC017 (**A**). HIV-1_IIIB_ was cultured in C8166 cells for 13 passages in the presence of increasing concentrations of the compound. Genotypic identification of ACC017-induced drug-resistant strains (**B**).

HIV-1 strains resistant to ACC017 were selected in C8166 cells by passaging the virus in the presence of increasing concentrations of ACC017. After 13 passages (Figure 6A), mutant viruses were harvested. The virus from the 13th passage was amplified, and its TCID_50_ was determined. This virus was named HIV-1_DRACC017_ and used for phenotypic drug resistance assays. As shown in Table 4, a more than 4000-fold reduction in inhibitory potency was observed for ACC017 against HIV-1_DRACC017_ compared with that against HIV-1_IIIB_, indicating substantially reduced activity of ACC017 against the resistant strain HIV-1_DRACC017_. DTG also showed a pronounced reduction in potency (>4237-fold). The decrease in potency was markedly less for 3TC (1.78-fold) and EFV (2.07-fold). The above results demonstrate that the ACC017-resistant strain was successfully induced, and subsequent experiments can be carried out.

In order to identify the mutation sites of the ACC017-induced drug-resistant strains, we amplified and sequenced the full-length integrase sequence (864 bp) of its drug-resistant strains. In this experiment, the cloning sequencing method was used. For each viral generation, 40 positive clones were selected for sequencing. After quality control, 30 sequences from each generation were chosen for sequence analysis. A series of mutation sites were discovered, as shown in Figure 6B. The results showed that the D232N and R263K mutations started to appear from the 2nd generation of the virus and persisted until the 13th generation. The higher the generation, the higher the frequency of these mutations. In each passage, 5–6 clones were identified that carried both the D232N and R263K mutations on the same genome. Other major integrase inhibitor resistance mutations, including H51Y, T66A, E92V, G118R, E138K, Y143C/H, P145S, S153F, N155S, and G163K, were all detected at the mutation sites of the in vitro-induced resistant strains of ACC017. However, their frequencies did not increase with the increase in drug concentration. In addition, the resistance mutations G140S/A/C/R and Q148H/R/K, which frequently occur in the first- and second-generation integrase inhibitors, were not detected in the resistant viruses induced in vitro by ACC017. No integrase inhibitor-related resistance mutations were observed in the co-cultured HIV-1_IIIB_ without the addition of ACC017.

According to the results of genotypic resistance analysis, we constructed the mutant strain NL4-3_IN-R263K_ with a mutation at the 263rd amino acid position of the integrase gene. The construction of the site-directed mutant strain was verified by sequencing. Then, we detected the inhibitory activities of ACC017 and the positive control drug against this virus (Table 4). ACC017 and DTG demonstrated mild resistance to the mutant strain. The EC_50_ values increased from 1.70 ± 0.47 nM to 28.09 ± 4.22 nM (Fold Change, FC = 16.52) for ACC017 and from 2.41 ± 0.43 nM to 32.21 ± 3.74 nM (FC = 13.37) for DTG, respectively. In contrast, the inhibitory activities of the control drugs 3TC and EFV against the mutant strain NL4-3_IN-R263K_ were comparable to those against the wild-type NL4-3, consistent with their non-integrase targeting mechanisms. Indeed, our sequencing data revealed mutations not only in integrase but also in other genomic regions. These findings suggest a substantial divergence from phenotypes associated with single-point mutations, indicating that the observed high-level resistance is likely due to mutations at multiple sites.

In addition, ACC017 and DTG effectively inhibited the replication of the INSTI-resistant strain pNL4-3_G140S/Q148H_ (Table 1). Compared to their activity against the wild-type NL4-3 strain (Table 4), the pNL4-3_G140S/Q148H_ mutant showed a 4.82-fold reduction in susceptibility to ACC017 (EC_50_: 1.70 nM vs. 8.19 nM) and a 5.59-fold reduction to DTG (EC_50_: 2.41 nM vs. 13.46 nM). These results indicate that although the HIV-1 integrase double mutant G140S/Q148H exhibits reduced susceptibility to ACC017 and DTG, both drugs retain comparable potency in inhibiting the replication of this drug-resistant strain.

## 4. Conclusions

Our study demonstrates that ACC017, a novel integrase inhibitor, exhibits potent anti-HIV-1 activity against laboratory-adapted strain, drug-resistant strains, and clinically isolated strains in different cell lines with low cytotoxicity. Its mechanism of action is confirmed to inhibit the integration of the viral genome into the host DNA. Furthermore, ACC017 shows synergistic effects with other antiretroviral drugs. In vitro resistance selection primarily yielded D232N and R263K mutations, without the emergence of common resistance mutations against first- and second-generation integrase inhibitors—namely G140S/A/C/R and Q148H/R/K. This distinct resistance profile suggests that ACC017 may remain active against some virus strains resistant to existing INSTIs, and that further testing will be required in the future. A study in healthy adults demonstrated that ACC017 has a favorable safety and tolerability profile following single-dose administration (CTR20240167). The clinical program is actively advancing, with a Phase II trial (CTR20250437) currently ongoing and a Phase III trial (CTR20253999) having recently initiated patient recruitment. These developments highlight its potential as a viable alternative in the future development of anti-HIV-1 drugs.

## Figures and Tables

**Table 1:** Anti-HIV-1 activities of ACC017 against laboratory-adapted strain, drug-resistant strains and clinical isolated strains.

Virus ^a^	Cell	CC_50_ (μM) ^b^ (Mean ± SD)		Testing Method	EC_50_ (nM) ^c^ (Mean ± SD)		SI ^d^	
		ACC017	DTG		ACC017	DTG	ACC017	DTG
HIV-1_IIIB_	C8166	20.37 ± 0.10	20.34 ± 0.07	Syncytia	0.59 ± 0.09	0.73 ± 0.02	34,525.42	27,863.01
HIV-1_IIIB_	C8166	20.37 ± 0.10	20.34 ± 0.07	p24	0.59 ± 0.09	0.64 ± 0.07	34,525.42	31,781.25
HIV-1_IIIB_ (20% HS) ^e^	C8166	/ ^f^	/	p24	8.28 ± 2.02	33.57 ± 5.90	/	/
HIV-1_IIIB_	MT4	24.80 ± 0.08	24.91 ± 0.01	MTT	51.47 ± 0.02	51.44 ± 0.14	496,000.00	498,200.00
					(pM)	(pM)		
HIV-1_4755-5_	C8166	20.37 ± 0.10	20.34 ± 0.07	p24	9.12 ± 2.31	9.96 ± 2.78	2233.55	2042.17
HIV-1_A17_	C8166	20.37 ± 0.10	20.34 ± 0.07	p24	8.51 ± 0.25	4.78 ± 5.13	2393.65	4255.23
pNL4-3_gp41(36G) V38A, N42T_	C8166	20.37 ± 0.10	20.34 ± 0.07	p24	0.34 ± 0.09	1.95 ± 0.12	59,911.76	10,430.77
HIV-1_RF/V82F/184V_	C8166	20.37 ± 0.10	20.34 ± 0.07	p24	1.45 ± 0.48	1.69 ± 1.22	14,048.28	12,035.50
pNL4-3_G140S/Q148H_	C8166	20.37 ± 0.10	20.34 ± 0.07	p24	8.19 ± 4.88	13.46 ± 8.41	2487.18	1511.14
HIV-1_WAN_	PBMC	15.24 ± 1.15	14.56 ± 3.62	p24	1.78 ± 0.29	8.23 ± 0.20	8561.80	1769.14
HIV-1_KIZ011_	PBMC	15.24 ± 1.15	14.56 ± 3.62	p24	0.33 ± 0.06	0.28 ± 0.03	46,181.82	52,000.00
HIV-1_TC-1_	PBMC	15.24 ± 1.15	14.56 ± 3.62	p24	0.11 ± 0.06	0.31 ± 0.18	138,545.45	46,967.74

^a^ Virus: Laboratory-adapted strain HIV-1_IIIB_; Drug-resistant strains HIV-1_4755-5_, HIV-1_A17_, pNL4-3_GP41(36G) V38A, N42T_, HIV-1_RF/V82F/184V_. and pNL4-3_G140S/Q148H_; Clinical isolated HIV-1 strains HIV-1_TC-1_, HIV-1_KIZ011_, and HIV-1_WAN_. ^b^ CC_50_: the 50% cytotoxicity concentration, as determined by the MTT method. ^c^ EC_50_: the 50% effective concentration, as determined by syncytium counting, p24 antigen ELISA, or the MTT assay. ^d^ SI: selectivity index, the ratio of CC_50_/EC_50_. ^e^ 20% HS: 20% human serum. Inactivated human serum was mixed with ACC017 or DTG to evaluate the effect of serum protein on drug activity. ^f^ /: a slash indicates undetected or uncalculated values. All data represent the mean ± SD for three independent replicate experiments.

**Table 2:** Inhibitory activity of ACC017 against HIV-1 integrase.

Compd.	IC_50_ (nM) (Mean, n = 4) ^a^	
	HIV-1 WT	HIV-1_N155H_
ACC017	9.19	9.91
DTG	13.60	9.26

^a^ IC_50_: concentration of compounds needed to prevent 50% of HIV-1 IN activity. All data represent the mean for four replicate wells.

**Table 3:** Effect of ACC017 in combination with anti-HIV-1 drugs on antiviral activity in vitro.

Drugs	Drug Type	Single Drug	DRI Value ^a^				CI Value ^f^	Description
			ED_50_^b^	ED_75_^c^	ED_90_^d^	ED_95_^e^		
ACC017 + ACC017	INSTIs	ACC017	1.56	1.77	2.06	2.32	0.95 ± 0.33	additive
	INSTIs	ACC017	4.48	3.46	2.77	2.42		
ACC017 + DTG	INSTIs	ACC017	3.65	2.89	2.45	2.27	1.57 ± 0.87	antagonism
	INSTIs	DTG	1.80	1.40	1.40	1.60		
ACC017 + 3TC	INSTIs	ACC017	3.04	5.25	9.07	13.15	0.54 ± 0.24	synergism
	NRTIs	3TC	4.89	6.21	7.90	9.30		
DTG + 3TC	INSTIs	DTG	2.34	3.00	4.01	4.98	0.51 ± 0.32	synergism
	NRTIs	3TC	3.73	5.03	6.79	8.33		
ACC017 + ACC007	INSTIs	ACC017	2.61	4.07	6.48	8.97	0.38 ± 0.12	synergism
	NNRTIs	ACC007	2.41	3.77	6.12	8.66		
DTG + ACC007	INSTIs	DTG	2.27	3.00	4.13	5.21	0.49 ± 0.22	synergism
	NNRTIs	ACC007	3.03	4.66	7.34	10.16		
ACC017 + FTC + TAF	INSTIs	ACC017	3.30	4.62	6.79	9.05	0.47 ± 0.18	synergism
	NRTIs	FTC	4.90	7.82	12.57	17.41		
	NRTIs	TAF	3.61	4.78	6.99	8.96		
DTG + FTC + TAF	INSTIs	DTG	2.46	3.25	4.37	5.41	0.61 ± 0.19	synergism
	NRTIs	FTC	4.27	7.15	12.20	17.77		
	NRTIs	TAF	3.10	4.40	6.33	8.18		

^a^ DRI: dose reduction index. ^b^ ED_50_: the 50% effective dose. ^c^ ED_75_: the 75% effective dose. ^d^ ED_90_: the 90% effective dose. ^e^ ED_95_: the 95% effective dose. ^f^ CI: combination index. Data represent the mean ± SD for three independent replicate experiments.

**Table 4:** Antiviral activity of ACC017, DTG, 3TC, and EFV against HIV-1 wild-type and mutant strains.

Compd.	Testing Method	EC_50_ (nM) ^a^		FC ^b^	EC_50_ (nM)		FC
		HIV-1_IIIB_	HIV-1_DRACC017_		NL4-3	NL4-3_IN-R263K_	
ACC017	p24	1.25 ± 0.48	>5000.00	>4000.00	1.70 ± 0.47	28.09 ± 4.22	16.52
DTG	p24	1.18 ± 0.15	>5000.00	>4237.29	2.41 ± 0.43	32.21 ± 3.74	13.37
3TC	p24	181.45 ± 41.48	322.32 ± 34.63	1.78	197.81 ± 100.51	180.05 ± 38.49	0.91
EFV	p24	0.60 ± 0.18	1.24 ± 0.22	2.07	1.51 ± 0.48	1.62 ± 0.31	1.07

^a^ EC_50_: the 50% effective concentration, as determined by HIV-1 p24 antigen ELISA. Data represent the mean ± SD for three independent replicate experiments. ^b^ FC: Fold Change, calculated as EC_50_ (HIV-1_DRACC017_)/EC_50_ (HIV-1_IIIB_) and EC_50_ (NL4-3_IN-R263K_)/EC_50_ (NL4-3).

## Data Availability

The data presented in this study are available in the present article.
